# Relationship Power, Antiretroviral Adherence, and Physical and Mental Health Among Women Living with HIV in Rural Kenya

**DOI:** 10.1007/s10461-022-03775-6

**Published:** 2022-08-24

**Authors:** Rachel L. Burger, Craig R. Cohen, A. Rain Mocello, Shari L. Dworkin, Edward A. Frongillo, Elly Weke, Lisa M. Butler, Harsha Thirumurthy, Elizabeth A. Bukusi, Sheri D. Weiser

**Affiliations:** 1 Department of Psychiatry and Behavioral Sciences, University of California, San Francisco, CA, USA; 2 Department of Obstetrics, Gynecology & Reproductive Sciences, University of California, San Francisco, CA, USA; 3 School of Nursing and Health Studies, University of Washington, Bothell, WA, USA; 4 Department of Health Promotion, Education, and Behavior, University of South Carolina, Columbia, SC, USA; 5 Centre for Microbiology Research, Kenya Medical Research Institute, Nairobi, Kenya; 6 Institute for Collaboration on Health, Intervention and Policy, University of Connecticut, Storrs, CT, USA; 7 Perelman School of Medicine, University of Pennsylvania, Philadelphia, PA, USA; 8 Department of Medicine, University of California, San Francisco, CA, USA

**Keywords:** Sexual relationship power, Mental health, Human immunodeficiency virus, AIDS, Kenya

## Abstract

Little is known about the association of gender-based power imbalances and health and health behaviors among women with HIV (WWH). We examined cross-sectional baseline data among WWH in a cluster-randomized control trial (NCT02815579) in rural Kenya. We assessed associations between the Sexual Relationship Power Scale (SRPS) and ART adherence, physical and mental health, adjusting for sociodemographic and social factors. SRPS consists of two subscales: relationship control (RC) and decision-making dominance. Women in the highest and middle tertiles for RC had a 7.49 point and 8.88 point greater Medical Outcomes Study-HIV mental health score, and a 0.27 and 0.29 lower odds of depression, respectively, compared to women in the lowest tertile. We did not find associations between SPRS or its subscales and ART adherence. Low sexual relationship power, specifically low RC, may be associated with poor mental health among WWH. Intervention studies aimed to improve RC among WWH should be studied to determine their effect on improving mental health.

## Resumen

Poco se sabe acerca de su asociación con los desequilibrios de poder basados en el género y los comportamientos de salud y salud entre las mujeres con Virus de Inmunodeficiencia Humana (VIH). Examinamos los datos de referencia transversales entre mujeres con VIH en un ensayo de control aleatorizado por grupos (NCT02815579) en las zonas rurales de Kenia. Evaluamos las asociaciones entre la Escala de Poder de Relación Sexual y la adherencia a la Terapia Antirretroviral (TAR), la salud física y mental, ajustando por factores sociodemográficos y sociales. La Escala de Poder de Relación Sexual consiste de dos subescalas: control de relaciones y dominio en la toma de decisiones. Las mujeres en los terciles más alto y mediano para control de relaciones tenían una puntuación de salud mental de 7.49 puntos y 8.88 puntos mayor en el Medical Outcomes Study HIV Health Survey (MOS)-HIV, y una puntuación de salud mental de 0.27 y 0.29 menores probabilidades de depresión, respectivamente, en comparación con las mujeres en el tercil más bajo. El bajo poder de relación sexual, específicamente el control de relaciones bajo, puede estar asociado con una salud mental deficiente entre las mujeres con VIH. Se deben estudiar estudios de intervención destinados a mejorar el control de relaciones entre mujeres con VIH para determinar su efecto en mejorar la salud mental.

## Introduction

Studies have shown that power inequality within heterosexually-active relationships is linked to poor sexual and reproductive health outcomes for women [1–3]. In the application of the Sexual Relationship Power Scale (SRPS) to HIV prevention research [4, 5], lower SRPS scores have been associated with higher sexual risk for HIV infection [1, 6]. Furthermore, gender-based power imbalance is a known risk factor for intimate partner violence [1, 3, 4, 7–9]. Among HIV positive women with low sexual relationship power, there is increased risk of malnutrition, specifically low Body Mass Index and low Mid-Upper Arm Circumference [7]. A recent study in rural Uganda that showed that low sexual relationship power contributed to depression among HIV-infected women [10]. Among women with HIV/AIDS (WWH), however, less is known about the effects of sexual relationship power on other health behaviors such as adherence to antiretroviral therapy (ART) and physical and mental health quality of life.

Adherence to ART is a critical determinant of HIV-1 RNA viral suppression and health outcomes [11–13], and an emerging literature shows that relationship factors may both interfere with and support adherence [14–16]. Partners may provide support for medication adherence by providing reminders and social support (instrumental, informational and emotional) [14–16]. Male partners are not always supportive of their partner’s medication adherence, particularly when there is a power imbalance within the relationship [14]. Sexual relationship power may also contribute to poor mental and physical health among WWH, which could further undermine ART adherence [10].

To understand the associations of sexual relationship power with ART adherence and physical and mental health among WWH in rural Kenya, we conducted a cross-sectional analysis of data collected in Shamba Maisha, a cluster randomized controlled trial. *Shamba Maisha* is a multisectoral agricultural and financial intervention trial to improve health outcomes among HIV-infected farmers in western Kenya (NCT02815579).

## Methods

### Participants

The study took place in Kenya within Kisumu, Migori, and Homa Bay counties and used baseline data collected between June 2016 and December 2017 as part of Shamba Maisha. Sixteen health facilities were randomized 1:1 to intervention or control arms. Inclusion criteria for the larger study included adults living with HIV between the ages of 18 and 60 years old who were receiving ART, who experienced food insecurity and/or malnutrition (BMI < 18.5) with access to farming land and surface water, and who agreed to save the down payment for a loan. All participants gave written informed consent prior to enrollment. Participants in the intervention facilities received trainings on sustainable farming practices and financial literacy, as well as an asset loan (~$150 USD) to purchase a water pump, seeds, fertilizer, and other farming inputs after making a down payment of approximately $9 USD.

### Data Collection

Interviewer-administered instruments were used to collect data on sexual relationship power, ART adherence, HIV disclosure, stigma, mental and physical health, economic and agriculture data, and other socio-demographic factors. Surveys and written consent forms were translated and administered by a Dholuo or Kiswahili speaker. Clinical data were abstracted from the medical records. We received ethical approval from the Kenya Medical Research Institute Scientific and Ethical Review Unit and the University of California San Francisco Institutional Review Board.

#### Measurements

Our primary explanatory variable, relationship power, was measured using the sexual relationship power scale (SPRS) [5], a 22-item validated scale that has been used in research conducted in black African populations [1, 4, 10, 17]. Questions were asked about participants’ current intimate relationship or the last one if they were not in a relationship. The SRPS contains two subscales: Relationship Control and Decision-Making Dominance. The Relationship Control subscale has fourteen questions rated on a 4-point Likert-type scale ranging from Strongly Agree (1) to Strongly Disagree (4) to assess the extent to which women can exert sexual and emotional autonomy (e.g., “My partner tells me who I can spend time with.”). The Decision-Making Dominance sub-scale measures the balance of decision-making power (1 = Your partner has more power; 2 = Both of you have equal power; 3 = You have more power). For example, one Decision-Making Dominance item asks “Who usually has more say about what you do together?” Responses are summed and normalized to a range of 1–4, with higher scores indicating greater relationship power. As suggested by Pulerwitz et al., [5] scale scores were split into tertiles representing ‘low’, ‘medium’ and ‘high’ power. Both subscales had good internal reliability (Relationship Control Cronbach’s alpha = 0.84, Decision-Making Dominance alpha = 0.78), as did the SRPS scale as a whole (Cronbach’s alpha = 0.86). Previous research on the SRPS subscales have also been mixed, with many authors omitting Decision-Making Dominance, and others showing that only the Relationship Control sub-scale influenced outcomes [1, 6]. A Systematic Review of the Psychometric Properties of the SRPS in HIV/AIDS Research found that the SRPS and Relationship Control subscale exhibited sound psychometric properties across multiple study populations and research settings. The Decision-Making Dominance subscale had relatively weak psychometric properties, especially when used with specific populations (i.e. younger age) and research settings [18].

Primary outcomes: ART adherence was measured with a visual analogue scale (VAS), a simple psychometric measurement tool using a continuous scale that has concordance with 3-day recall and unannounced pill counts [19–21]. We dichotomized adherence as ≥ 95% of prescribed doses taken in the prior 30 days compared to < 95% using the VAS [21], based on literature linking 95% self-reported adherence to virologic outcome for patients with HIV [11]. Physical and mental health status were assessed with the Medical Outcomes Study (MOS)-HIV health-related quality-of-life subscales, physical health summary score (PHS) and mental health summary score (MHS). Both subscales are continuous with a range of 0–100. The MOS-HIV reliability and validity has been well documented [22, 23], and adapted for use in East Africa [24]. Depression symptom severity was measured with the Hopkins Symptom Checklist Depression Scale (HSCL-D) [25, 26]. A value of ≥ 1.75 on the HSCL-D is consistent with screening positive for symptoms of depression, thus we created a dichotomous variable using that cut-off.

Covariates: We chose potential socioeconomic and clinical confounders based on literature and theory including age, any secondary education, marital status (single, married, widowed, and separated), household wealth (quintiles), hazardous drinking as measured by the AUDIT-C [27], and duration of ART [28, 29].

### Statistical Analysis

We performed a cross-sectional baseline analysis among women participants to determine the association of sexual relationship power with ART adherence and physical and mental health status. We fitted multivariable logistic regression models to test for associations between the full scale and two subscales and excellent self-reported ART adherence and depression symptom severity. We split the scales because the Decision-Making Dominance has consistently lower reliability, as described above. We ran multivariable linear regression models to assess associations between relationship power and PHS and MHS scales. For each outcome, we fit one model using overall SRPS as the primary predictor and a separate model that contained the Relationship Control and Decision-Making Dominance subscales, to evaluate whether the two domains were differentially associated with the outcomes of interest. We evaluated the associations between all candidate covariates and our primary independent and dependent variables. We adjusted all models for continuous age and years on ART, marital status (married vs. not), educational attainment (secondary or higher vs. primary or lower), wealth index (quintiles), and hazardous drinking. All models accounted for clustering at the health facility level using a mixed model with health facility as the random effect. Analyses were performed using SAS 9.4 (SAS Institute Inc., Cary, NC).

## Results

Three hundred and eighty two WWH were analyzed. From the larger study, 14 were excluded due to incomplete SRPS data. The median age was 38 years (IQR 31–44 years), 60.7% were married, and 20.4% had some secondary education (Table 1). The median Relationship Control score was 2.6 with a range of 1.1–4.0. In the bivariate model (Table 2), women with the highest and middle tertiles for Relationship Control had an 8.35 point (p < 0.001) and 6.83 point (p < 0.001) higher mental health score (range 0–100), respectively, compared to women in the lowest tertile. Women with the highest and middle tertiles for Relationship Control also had a 0.38 (p = 0.001) and 0.32 (p < 0.001) lower odds of screening positive for depression, respectively, compared to women in the lowest tertile.

Relationship Control was also associated with depression and MOS-HIV mental health in the multivariable models (Table 3). Women in the highest and middle tertiles for Relationship Control had a 8.88 point (p < 0.001) and 7.49 point (p < 0.001) greater mental health score (range 0–100), respectively, compared to women in the lowest tertile. Women in the highest and middle tertiles for Relationship Control had a 0.29 (p < 0.001) and 0.27 (p < 0.001) lower odds of depression, respectively, compared to women in the lowest tertile. Women in the highest tertile of Relationship Control had 4.11 higher points physical health status sub-scale of the MOS-HIV when compared with women with the lowest tertile, that was not significant (p = 0.098). Relationship Control was not associated with ART adherence. The proportion of WWH achieving ≥ 95% ART adherence was similar across Relationship Control tertiles (from 0.69 to 0.74). Decision-Making Dominance was not associated with any of the outcomes (Table 3).

Of the 382 women analyzed at baseline, 280 (73%) were in a relationship and 102 (27%) were not, with 83% of the latter being widows. Women who were not in a relationship were asked about their last relationship. We ran a sensitivity analysis restricted to women who reported being in a current relationship to assess whether relationship recency had a differential effect on our outcomes of interest. We found no differences in the direction, magnitude, or significance of the associations we reported for the full analytic sample. Results not shown.

## Discussion

We found that women with higher sexual relationship power were less likely to meet criteria for probable depression compared to women with low relationship power. These results were supported by a study in rural Uganda that showed that low sexual relationship power contributed to depression among HIV-infected women [10]. We found higher levels of probable depression among this population (44.8%) compared to the Ugandan WWH (23.7%) [10].

This study also examined the effect of relationship power on ART adherence, physical health, and mental health among WWH. Quality of life and wellbeing, as measured by the MOS-HIV scores (range 0–100) were higher in this population compared to a mixed-gender HIV outpatients study in East Africa (mental health score 59.2 in our sample compared to 46.2, and physical health (83.1 in our sample compared to 44.9) [30]. Women with higher sexual relationship power had better mental health status and tended to have better physical health compared to women with low relationship power. However, cross-sectional data preclude making causal conclusions. Relationship power was not associated with ART adherence in the current study. This could be due to a relatively high percentage (71.7%) of participants that achieved ≥ 95% adherence. This also could also be due to the reliance on self-reported adherence, which is an imperfect measure [31] and may mask underlying associations between Relationship Control and adherence.

The association between physical health and sexual relationship power was stronger with the Relationship Control sub-scale compared to the Decision-Making Dominance sub-scale, though effects were not statisitically significant. These results are consistent with previous literature [18] and together suggest that Relationship Control may be a more sensitive predictor of poor physical and mental health risk in this population.

Our study had several limitations. First, our sample consisted of HIV-positive women on ART who mainly resided in rural Kenya and were food insecure; therefore, our findings may not be generalizable. Second, our measure of probable depression does not provide a diagnosis of major depressive disorder and the relationship of mental health and sexual relationship power is likely bi-directional. Previous theory and literature have suggested several plausible mechanisms through which low sexual relationship power could lead to depression [10]. At the same time, it is certainly possible that people who are depressed are more likely to over report low sexual power. In-depth, qualitative research could further delineate the mechanisms through which sexual power may affect mental health. Our findings could imply that low Relationship Control among WWH may increase their risk of poor mental health, or that poor mental health among WWH may lead to reduced Relationship Control. Longitudinal studies are needed to confirm the direction of these associations.

Interventions to improve mental health among HIV-positive women should consider strategies that improve women’s Relationship Control and improve partner relationship equality. A multi-level intervention may be required to address factors such as access to HIV treatment, social support, stigma and discrimination, disclosure, poverty, food security, and land security. Structural strategies such as economic empowerment and gender transformative interventions [32] could be adapted or intensified for WWH. Interventions focused on men and gender transformative interventions have also shown promises and limitations [33, 34]. At the relationship level, couples-based interventions may provide opportunities to address gendered power and relationship dynamics from both partners’ perspectives [35].

## Conclusions

In conclusion, Relationship Control in a sample of WWH in Kenya was strongly associated with symptoms of depression and worse mental health status. Longitudinal studies are needed to assess the direction of these associations. Interventions designed to enhance the intimate relationships that shape women’s overall health and well-being may have the potential to improve outcomes of women suffering from the syndemic of HIV/AIDS and poor mental health.

## Figures and Tables

**Table 1:** Descriptive statistics for cohort of HIV-positive women in rural Kenya

	All participants (N = 382) N (%) or median (IQR)
Socio-demographics characteristics	
Age (y)	38 (31, 44)
Married	60.7%
Education	
None or primary	79.6%
Secondary	20.4%
Household characteristics	
Improved water source	45.9%
Improved sanitation facility	41.2%
Finished floor composition	29.8%
Social and behavioral variables	
Hazardous drinking (AUDIT-C)	4.7%
Social support score^a^	2.0 (1.7, 2.4)
Anticipated stigma score^b^	2.0 (1.3, 2.1)
Enacted stigma score	1.0 (1.0, 1.1)
Internalized stigma score	2.0 (1.7, 2.7)
Disclosed HIV to primary partner	94.0%
Visual adherence scale (VAS) ≥ 95%	71.7%
Clinical characteristics	
Any AIDS-defining condition	4.2%
CD4+ count, (% ≤ 200 cells/|μL)	2.4%
HIV viral load ≥ 200 cells/mm^3^	18.1%
MOS HIV physical health scale^c^	83.1 (68.9, 87.7)
MOS HIV mental health scale^c^	59.2 (46.9, 70.1)
Probable depression (HSCL-D ≥ 1.75)	44.8%
Time on current ART regimen (years)	4.7 (2.6, 6.9)
Sexual Relationship Power Scale (SPRS)	
Sexual relationship power (SPRS), full scale score^d^	2.2 (1.9, 2.5)
Decision-making dominance (DMD), subscale score^d^	1.9 (1.6, 2.5)
Relationship Control (RC), subscale score^d^	2.6 (2.2, 2.9)

*HSCL* Hopkins Symptomatic Check List; *AUDIT-C* Alcohol Use Disorders Identification Test

a Range: 1–4 (lower = more social support)

b Range: 1–5 (lower = less stigma)

c Range: 0–100 (higher = better health)

d Range: 1–4 (higher = greater control)

**Table 2:** Bivariable analysis of relationship power, adherence, and physical and mental health among HIV-positive women in rural Kenya

	Visual Adherence Scale ≥ 95%		MOSHIV Physical Health Score		MOSHIV Mental Health Score		Binary depression	
				
	OR (95% CI)	p value	β (SE)	p value	β (SE)	p value	OR (95% CI)	p value
Sexual Relationship Power Scale								
Low	*Referent*		*Referent*		*Referent*		*Referent*	
Medium	1.17 (0.68, 2.03)	0.679	0.58 (2.13)	0.784	4.30 (1.88)	0.023	0.56 (0.32, 0.96)	0.034
High	1.29 (0.74, 2.23)	0.352	1.05 (2.12)	0.620	4.60 (1.88)	0.015	0.60 (0.35, 1.03)	0.063
Relationship Control subscale								
Low	*Referent*		*Referent*		*Referent*		*Referent*	
Medium	1.23 (0.72, 2.11)	0.469	1.05 (2.10)	0.616	6.83 (1.82)	< 0.001	0.32 (0.18, 0.55)	< 0.001
High	1.07 (0.61, 1.88)	0.73	2.68 (2.23)	0.229	8.35 (1.93)	< 0.001	0.38 (0.21, 0.67)	0.001
Decision-Making Dominance subscale								
Low	*Referent*		*Referent*		*Referent*		*Referent*	
Medium	0.92 (0.53, 1.58)	0.619	1.13 (2.16)	0.602	1.27 (1.93)	0.51	0.93 (0.54, 1.60)	0.806
High	1.36 (0.78, 2.37)	0.264	− 2.17 (2.09)	0.302	1.58 (1.87)	0.400	0.93 (0.55, 1.57)	0.786

**Table 3:** Multivariable analysis of relationship power, adherence, and physical and mental health among HIV-positive women in rural Kenya

	Visual Adherence Scale ≥ 95%		MOSHIV Physical Health Score		MOSHIV Mental Health Score		Binary depression	
				
	AOR	p value	Adjusted β	p value	Adjusted β	p value	AOR	p value
Relationship Control subscale								
Low	*Referent*		*Referent*		*Referent*		*Referent*	
Medium	1.17	0.607	1.323	0.559	7.489	< 0.001	0.268	< 0.001
High	0.858	0.644	4.108	0.098	8.881	< 0.001	0.288	< 0.001
Decision-Making Dominance subscale								
Low	*Referent*		*Referent*		*Referent*		*Referent*	
Medium	0.921	0.791	0.155	0.947	− 2.082	0.305	1.567	0.158
High	1.329	0.366	− 3.108	0.177	− 2.003	0.318	1.568	0.155
^^^Age	1.028	0.065	− 0.2744	0.010	− 0.011	0.902	1.025	0.087
^^^Wealth (quintiles)								
1st (lowest)	*Referent*		*Referent*		*Referent*		*Referent*	
2nd	*1.157*	*0.679*	*− 0.900*	0.740	− 0.418	0.860	0.933	0.852
3rd	*1.487*	*0.297*	*2.565*	0.370	5.402	0.031	0.523	0.101
4th	*1.315*	*0.463*	−*1.970*	0.387	3.996	0.106	0.760	0.485
5th (highest)	*1.991*	*0.090*	*− 0.756*	0.808	4.893	0.074	0.574	0.196
Marital status^a^								
Single, widowed, divorced	*Referent*		*Referent*		*Referent*		*Referent*	
Married/in a partnership	*0.813*	*0.429*	*0.111*	0.947	− 0.118	0.943	1.309	0.300
Educational attainment^a^								
Primary or less	*Referent*		*Referent*		*Referent*		*Referent*	
Secondary or higher	*0.912*	*0.764*	*− 0.188*	0.947	− 2.259	0.253	1.653	0.094
Hazardous drinking^a^	*0.856*	*0.784*	*9.30*	0.025	0.840	0.815	0.760	0.617
Length of time on ART (years)^a^	*0.961*	*0.380*	*0.150*	0.649	0.273	0.342	0.987	0.761

a Covariates based on the subscale analysis
